# Association between Obese Phenotype and Mildly Reduced eGFR among the General Population from Rural Northeast China

**DOI:** 10.3390/ijerph13060540

**Published:** 2016-05-27

**Authors:** Shasha Yu, Hongmei Yang, Xiaofan Guo, Liqiang Zheng, Yingxian Sun

**Affiliations:** 1 Department of Cardiology, The First Hospital of China Medical University, 155 Nanjing North Street, Heping District, Shenyang 110001, China; yidasasa047717@126.com (S.Y.); yanghongmei047717@126.com (H.Y.); guoxiaofan047717@126.com (X.G.); 2 Department of Clinical Epidemiology, Shenjing Hospital of China Medical University, Shenyang 110003, China; zhengliqiang047717@126.com

**Keywords:** eGFR, mildly, obese phenotype, rural

## Abstract

Obesity contributes to reduced kidney function; however, whether this is due to obesity itself or the metabolic abnormalities that accompany it is unclear. Besides, most previous studies enrolled participants with moderate or severe stage of chronic kidney disease. In the present study, we aim to investigate the possible relationship between obesity, metabolic abnormalities and mildly reduced estimated glomerular filtration rate (eGFR). A total of 11,127 Chinese participants (age ≥ 35 years) were enrolled in a survey conducted from January 2012 to August 2013. eGFR 60–90 mL/min/1.73 m^2^ was defined as mildly reduced eGFR. Obese phenotype was divided into four types: metabolically healthy non-obese (MHNO), metabolically healthy obese (MHO), metabolically abnormal non-obese (MANO) and metabolically abnormal obese (MAO). Among all participants, 1941 (17.4%) of them had mildly reduced eGFR (16.7% for men and 18.1% for women, *p* = 0.025). The prevalence of obese phenotype was 22.5% for MHNO, 9.1% for MHO, 32.1% for MANO and 36.4% for MAO. The prevalence of mildly reduced eGFR was 9.0% among MHNO, 7.0% among MHO, 22.6% among MANO and 20.7% among MAO (*p* < 0.001). Multivariate logistic regression analysis revealed that obese phenotype did not statically contributed to mildly reduced eGFR (MHO: OR = 1.107, *p* = 0.662; MANO: OR = 0.800, *p* = 0.127; MAO: OR = 1.119, *p* = 0.525). However, gender (OR = 1.475, *p* < 0.001), aging (OR = 1.283, *p* < 0.001), dyslipidemia (OR = 1.544, 95%CI: 1.315, 1.814, *p* < 0.001) and hyperglycemia (OR = 1.247, 95%CI: 1.068, 1.455, *p* = 0.005) was associated with increased risk of mild reduced eGFR. Among the general population from rural Northeast China, mildly reduced eGFR was associated with metabolic disorders like dyslipidemia and hyperglycemia, but not obesity.

## 1. Introduction

Previous studies showed that overweight and obesity were associated with higher risk of chronic kidney disease (CKD) [1,2,3]. Some of them used body mass index to define overweight or obesity while others used waist circumference or waist-to-hip ratio [2]. Regardless of the differences of definition, they all came to the same conclusion that increasing obese measurement was associated with a reduction in estimated glomerular filtration rate (eGFR) for both men and women [2,3]. However, most of the previous study defined CKD as eGFR less than 60 mL/min/1.73 m^2^. Currently, there is lack of data about the relationship between obese phenotype and mildly reduced eGFR among the general population, especially from rural areas. 

A recent meta-analysis enrolled 11 studies that reported that Metabolic syndrome (MetS) was significantly associated with the development of eGFR less than 60 mL/min/1.73 m^2^ [4]. Similarly, Song and colleagues conducted a study of 75,468 urban Chinese and claimed that, in addition to obesity, metabolic abnormalities like elevated blood pressure, high triglycerides (TG) and impaired fasting glucose were related to the reduced GFR [5]. Overweight or obese subjects are usually accompanied by other metabolic disorders like hypertension, type 2 diabetes and lipid profile disorders. Both metabolic abnormalities and obesity contributed to decreased eGFR; it was hard to determine which one played the key role in decreasing eGFR. There have been many previous studies intending to figure out the possible relationship between metabolic profile and eGFR, but there have been inconsistent results. Some studies claimed that a healthy metabolic profile did not protect obese adults from incident CKD but others concluded that metabolic health obesity was not associated with higher risk of incident CKD. The aim of the present study was to clarify the possible relationship between metabolic abnormalities, obesity and decrease eGFR. In order to clarify the possible relationship between metabolic abnormalities, obesity and decrease eGFR, we redefined the criteria of obese phenotypes, including factors such as: metabolically health non-obese (MHNO), metabolically healthy obese (MHO), metabolically abnormal non-obese (MANO) and metabolically abnormal obese (MAO) [6].

## 2. Method

### 2.1. Study Population

**Figure 1:**
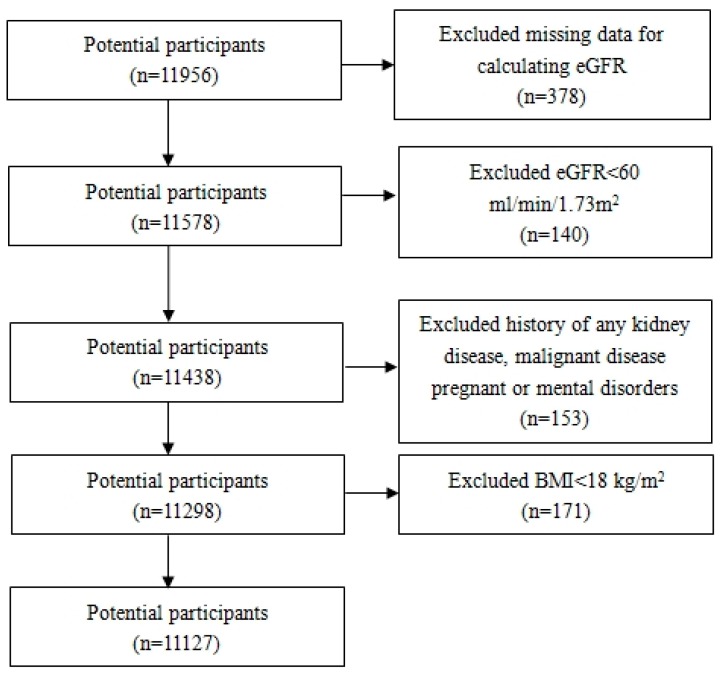
Flow diagram for study design showing inclusion of participant in analyses. BMI: body mass index; eGFR: estimated glomerular filtration rate.

Liaoning Province is located in Northeast China. From January 2012 to August 2013, a representative sample (age ≥ 35 years) was selected to describe the prevalence, incidence and natural history of cardiovascular risk factors in rural areas of Liaoning Province. The study adopted a multi-stage, stratified random cluster-sampling scheme. In the first stage, three counties (Dawa, Zhangwu and Liaoyang) were selected from the eastern, southern, and northern region of Liaoning Province. In the second stage, one town was randomly selected from each county (a total of three towns). In the third stage, 8–10 rural villages from each town were randomly selected (a total of 26 rural villages). Participants who were pregnant, or had a malignant tumor or mental disorder were excluded. All the eligible permanent residents aged ≥35 years from each village were invited to attend the study (a total of 14,016 participants). Of those, 11,956 participants agreed and completed the study to give a response rate of 85.3%. The study was approved by the Ethics Committee of China Medical University (Shenyang, China, AF-SDP-07-1, 0-01). All procedures were performed in accordance with the ethical standards. Written consent was obtained from each participant, after they had been informed of the objectives, benefits, medical items and confidentiality agreement of personal information. If the participants were illiterate, we obtained written informed consents from their close relatives. Figure 1 shows the flow chart for inclusion and exclusion of subjects. Finally, 11,127 participants (5221 men and 5906 women) were eligible for the study.

### 2.2. Data Collection and Measurements

Data were collected during a single clinical visit by cardiologists and trained nurses using a standard questionnaire by face-to-face interview. Before the survey was performed, we invited all eligible investigators to attend the organized training. The training contents included: the purpose of this study, ways to administer the questionnaire, standard method of measurement, importance of standardization, and study procedures. A strict test was evaluated after this training; only those who scored perfectly on the test could become investigators. During data collection, our inspectors had further instructions and support.

According to American Heart Association protocol, blood pressure (BP) was measured three times at 2-min intervals after at least 5 min of rest using a standardized automatic electronic sphygmomanometer (HEM-907; Omron Healthcare, Kyoto, Japan), which had been validated according to the British Hypertension Society protocol [7]. The participants were advised to avoid caffeinated beverages and exercise for at least 30 min before the measurement. During the measurement, the participants were seated with their arms supported at the level of the heart. The mean of three BP measurements was calculated and used in all analyses.

Weight and height were measured to the nearest 0.1 kg and 0.1 cm, respectively, with the participants wearing light-weight clothing and without shoes. Waist circumference (WC) was measured at the umbilicus using a non-elastic tape (to the nearest 0.1 cm), with the participants standing at the end of normal expiration. Body mass index (BMI) was calculated as the weight in kilograms divided by the square root of the height in meters.

Fasting blood samples were collected in the morning after at least 12 h of fasting. Blood samples were obtained from an antecubital vein into Vacutainer tubes containing ethylenediaminetetraacetic acid (EDTA). Fasting plasma glucose (FPG), total cholesterol (TC), low-density lipoprotein cholesterol (LDL-C), high-density lipoprotein cholesterol (HDL-C), triglycerides (TGs) and other routine blood biochemical indexes were analyzed enzymatically using an autoanalyzer. All laboratory equipment was calibrated, and blinded duplicate samples were used for these analyses. The eGFR based on creatinine level was calculated by Chronic Kidney Disease Epidemiology Collaboration (CKD-EPI) equation which was shown in Table 1 [8].

### 2.3. Definitions

According to the World Health Organization Asia Pacific guidelines, BMI ≥ 25 kg/m^2^ was defined as obesity [9]. MHO was defined as those who were only obese and had no other conditions. 

Metabolic abnormalities include: Hyperglycemia: FPG ≥ 6.1 mmol/L or previously diagnosed type 2 diabetes and receiving treatment; Hypertension: systolic blood pressure ≥140 mmHg, diastolic blood pressure ≥ 90 mmHg, or reported use of a medication for hypertension; Dyslipidemia: TG level >1.7 mmol/L, and/or HDL-C level <0.9 mmol/L (men) or <1.0 mmol/L (women) [10]. Obese residents had no other metabolic abnormalities and so were defined as MHO. Those who were not obese and had no metabolic abnormalities were MHNO. MANO or MAO subjects were defined as those who were not obese or obese and had at least one other metabolic abnormality. Normal eGFR was defined as those with eGFR ≥ 90 mL/min/1.73 m^2^ and eGFR 60–90 mL/min/1.73 m^2^ was defined as those with mildly reduced eGFR.

### 2.4. Statistical Analysis

Descriptive statistics were calculated for all the variables, including continuous variables (reported as mean values and standard deviations) and categorical variables (reported as numbers and percentages). The differences between groups were evaluated using ANOVA (LSD) for continuous data and Chi-square test for categorical data. Multivariate logistic regression analyses were used to identify odds ratios (ORs) for mildly reduced eGFR in MHO, MANO and MAO groups while the MHNO group was the reference group. All the statistical analyses were performed using SPSS version 17.0 software (SPSS Inc., Chicago, IL, USA) and *p*-values less than 0.05 were considered statistically significant.

## 3. Results

### 3.1. Characteristics of MHNO, MHO, MANO and MAO Subjects

The characteristics of all the participants are shown in Table 2. The proportion of MHNO, MHO, MANO and MAO subjects was 22.5% (2500/11,127), 9.1% (1009/11,127), 32.1% (3567/11,127) and 36.4% (4051/11,127), respectively. Compared with MHNO subjects, the MHO group had significantly higher metabolic factors, including BMI, WC, systolic BP, diastolic BP, TG, and HDL-C. Similarly, almost all the variables were significantly different between the MANO and MAO group except for eGFR. Surprisingly, a marked difference was not observed in FBG and creatinine between MHNO and MHO and in eGFR between MHNO and MHO and between MANO and MAO. There was a marked difference in the prevalence of mildly reduced eGFR between these four groups. In the MHNO and MHO group, only 9.0% and 7.0% had mildly reduced eGFR. In the contrast, 22.6% and 20.7% of the MANO and MAO group had mildly reduced eGFR.

### 3.2. Characteristics of Obese Phenotype and Mildly Reduced eGFR According to Gender and Age

**Figure 2:**
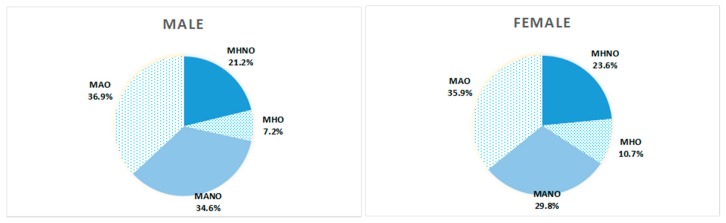
Prevalence of different obese phenotypes among rural Chinese resident by different genders.

**Figure 3:**
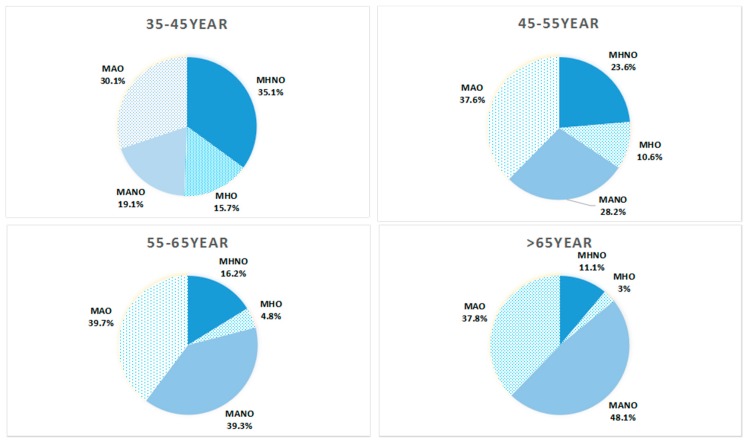
Prevalence of different **o**bese phenotypes among rural Chinese according to different age groups.

Figure 2 showed that MAO was the most prevalent obese phenotype among both women and men while MHO had the lowest proportion of all the phenotypes. In Figure 3, among different age groups, MNNO accounted for the largest part among 35–45 years group while the prevalence of MANO was highest among those in the >65 year group. Among the 45–55 year and 55–65 year groups, the rates of MAO were the highest.

**Figure 4:**
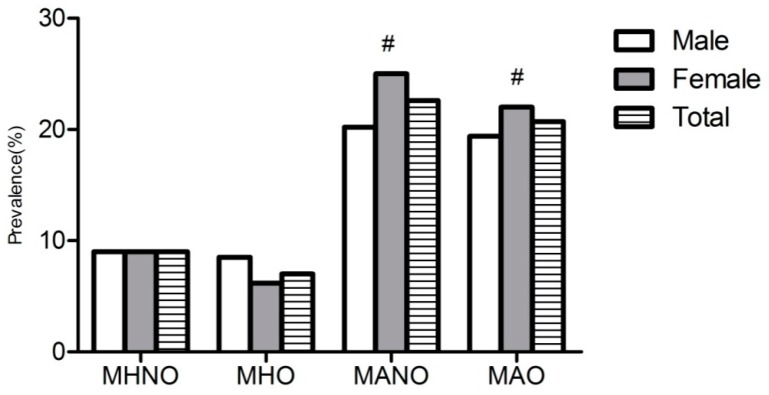
Prevalence of mild reduced eGFR among males and females in different obese phenotypes. # means compared with males, females have significantly higher prevalence of mild reduced eGFR.

Figure 4 showed that the prevalence of mildly reduced eGFR among different obese phenotypes according to gender. It showed that mildly reduced eGFR significantly increased among those MANO and MAO subjects. Among MANO (*p* < 0.001) and MAO (*p* = 0.025), women had a significantly higher prevalence of reduced eGFR then men.

### 3.3. Logistic Regression Analysis of the Association between Different Obese Phenotypes and Mildly Reduced eGFR

We estimated the ORs for mildly reduced eGFR in MHO, MANO and MAO groups using the MHNO group as the reference group and then calculating substrate by gender (Table 3). In model 1, MAO (OR 1.604, *p* < 0.001) subjects had significantly increased risk of mildly reduced eGFR. While further analysis showed gender difference, we found that this relationship existed only among men but not women. In order to explore the role of obesity in this phenomenon, we adjusted for BMI and WC in model 2. Increased ORs were also observed in MAO (OR 1.496, *p* = 0.005) among men only. To further analyze the role of metabolic abnormalities in this relationship, we adjusted for hypertension, hyperglycemia, and dyslipidemia in model 3. MAO (OR 1.119, *p* = 0.525) subjects, either men or women, would not present increased risk of mildly reduced eGFR anymore. Conversely, hyperglycemia (OR 1.247, *p* = 0.005) and dyslipidemia (OR 1.544, *p* < 0.001) but not hypertension (OR 1.028, *p* = 0.790), significantly increased the risk of mildly reduced eGFR. It is worth mentioning that MHO did not increase the risk of mildly reduced eGFR in all three models and both BMI and WC were not associated with the risk of mildly reduced eGFR.

## 4. Discussion

Due to the lack of uniform definition of obese phenotype, in our study, we used the criteria that is relatively widely used in the previous study [11]. With this criterion, the prevalence of MHNO, MHO, MANO and MAO were 22.5%, 9.1%, 32.1% and 36.4%, relatively. Meanwhile, the prevalence of mildly reduced eGFR was 9.0% among MHNO, 7.0% among MHO, 22.6% among MANO and 20.7% among MAO (*p* < 0.001). Multivariate analysis revealed that no obese phenotype contributed to mildly reduced eGFR. However, gender (OR = 1.475, *p* < 0.001), increasing age (OR = 1.283, *p* < 0.001) and metabolic abnormities like dyslipidemia (OR = 1.544, 95%CI: 1.315, 1.814, *p* < 0.001) and hyperglycemia (OR = 1.247, 95%CI: 1.068, 1.455, *p* = 0.005) were associated with increasing risk of mildly reduced eGFR.

Many previous studies that intend to estimate the association between obesity and CKD end up with conflicting results. Some claimed that high BMI contributed to the development of CKD while others suggested that, after further adjustment, the relationship between obesity and CKD was attenuated [12,13]. In the present study, after adjusting for possible confounders, we found that none of the obese phenotypes were associated with mildly reduced eGFR. Those with the conclusion that obesity was irrelevant to CKD thought that obesity was associated with an adverse health outcome like CKD largely due to its numerous metabolic complications like type 2 diabetes, hypertension and dyslipidemia [14,15]. Therefore, in a recent study, the definition of metabolic health obesity was identified to further investigate the association between obesity and mildly reduced eGFR. MHO was characterized by a lower frequency of diabetes, hypertension and dyslipidemia [16,17]. Chang Hee Jung and colleagues reported that the MHO group had significantly higher risk of CKD than the MHNO group [18]. It seemed that obesity itself might contribute to the increasing risk of CKD without the coexistence of metabolic disorders. The possible mechanisms to explain why obesity might cause decreased kidney function were still not well clarified. It might be mediated through multiple biologic mechanisms, including several hemodynamic, hormonal and inflammatory processes which cause intra-glomerular hypertension and finally result in glomerular damage [19,20]. Besides, oxidative stress and endothelial dysfunction also play a role in this relationship [21]. However, other studies claimed that MHO phenotype was not associated with higher risk of CKD [19]. Data from our study also confirmed that the obese phenotype was not associated with mildly reduced kidney function. The possible explanations for this might be, first, in our study, we estimated the mildly decreased kidney function (60–90 mL/min/1.73 m^2^) while most of the previous studies enrolled the severe CKD (<60 mL/min/1.73 m^2^). Secondly, the various definition of MHO resulted in the heterogeneity of the obese phenotype [22,23,24]. Furthermore, in our study, we did not clarify the existence of chronic kidney diseases among the enrolled participants. Many previous studies found that residents with chronic kidney disease might have lower muscle mass and dietary protein intake than healthy subjects. Thus, the relationships observed in the populations that had chronic kidney disease might differ from healthy populations, leading to increased errors when estimating using equations [25]. 

In this study, dyslipidemia and hyperglycemia was associated with increasing risk of mildly reduced eGFR in the fully adjusted model (model 3) among both women and men. The relationship between hyperglycemia/diabetes and reduced eGFR had been extensively studied [26,27]. Our study once again confirmed that hyperglycemia was associated with higher risk of mildly reduced eGFR among the general population in rural China (OR = 1.265 for women and OR = 1.250 for men, all *p* < 0.05). As for dyslipidemia, there was growing evidence that confirmed that lipid metabolic abnormalities contributed to the progression of renal deficiency [28,29]. Chen SC and colleagues conducted a study to estimate the relationship between dyslipidemia and renal outcomes in patients with moderate to advanced CKD and found that certain levels of dyslipidemia were independently associated with renal replacement therapy and rapid renal progression in CKD stage 3–5 [30]. A recent study also reported that dyslipidemia increased the incidence of CKD in middle-aged and elderly Chinese residents. Meanwhile, dyslipidemia played an important role in reducing eGFR and albuminuria [31]. However, the underling mechanisms existed in this relationship were not completely clarified. Aggravation of atherosclerosis of renal microcirculation, injury to glomerular capillary endothelial and mesangial cells, stimulation of inflammation and fibrogenesis by cytokines and growth factors might partially explain this association [32,33]. 

There are some strengths in our study. First, the present study enrolled a representative and large sample of the rural Chinese population. Second, we used the new CKD-EPI equation to estimate GFR, which has been proved more accurate and precise than the MDRD study and Cockcroft-Gault equations [34]. However, there were still some limitations in the present study. First, due to the cross-sectional design of our study, we cannot make inferences about causality. Second, until recently, there was a lack of unique definition of MHO phenotype which might cause various relationships between obese phenotypes and reduced eGFR. Third, reduced eGFR was based on a single assessment of blood samples, which may introduce error. Fourth, in the adjusted model we did not include some factors that might affect eGFR like uric acid and some medicine which might affect the accuracy of the results. Finally, it was not accurate to estimate GFR through the CKD-EPI equation. 

## 5. Conclusions 

In the present study, no significant association was confirmed between the obese phenotype and mildly reduced eGFR. However, we found that hyperglycemia and dyslipidemia were associated with reduced eGFR in both men and women from rural China. This is the first report regarding the contribution of metabolic abnormalities but not obesity to the early stage of CKD among the general population. Therefore, an early renal function screen was recommended among the subjects with dyslipidemia and hyperglycemia, especially women and aging subjects.

## Figures and Tables

**Table 1:** The definition of estimated glomerular filtration rate (eGFR) based on creatinine level was calculated by Chronic Kidney Disease Epidemiology Collaboration.

Sex	Scr (μmol/L)	eGFR (mL/min/1.73 m^2^)
Female	≤62	144 × (Scr/62)^−0.329^ × (0.993) ^age^
	>62	144 × (Scr/62)^−1.209^ × (0.993) ^age^
Males	≤80	144 × (Scr/62)^−0.411^ × (0.993) ^age^
	>80	144 × (Scr/62)^−1.209^ × (0.993) ^age^

**Table 2:** Characteristics of metabolically healthy non-obese (MHNO), metabolically healthy obese (MHO), metabolically abnormal non-obese (MANO) and metabolically abnormal obese (MAO) subjects.

Characteristics	MHNO (*n* = 2500, 22.5%)	MHO (*n* = 1009, 9.1%)	MANO (*n* = 3567, 32.1%)	MAO (*n* = 4051, 36.4%)	*p* Value ^a^
Female (%)	1392 (55.7)	632 (62.6)	1759 (49.3)	2123 (52.4)	<0.001
Age (years)	49.60 ± 9.54	48.11 ± 8.75 ^b^	56.79 ± 10.34 ^b,c^	54.26 ± 9.90 ^b,c,d^	<0.001
BMI (kg/m^2^)	22.05 ± 1.74	27.45 ± 2.65 ^b^	22.57 ± 1.68 ^b,c^	28.10 ± 2.64 ^b,c,d^	<0.001
WC (cm)	75.07 ± 6.39	86.41 ± 7.59 ^b^	78.23 ± 6.87 ^b,c^	90.15 ± 7.89 ^b,c,d^	<0.001
Current smoking (%)	920 (36.8)	258 (25.6)	1466 (41.1)	1271 (31.4)	<0.001
Current drining (%)	523 (20.9)	183 (18.1)	919 (25.8)	934 (23.1)	<0.001
Systolic BP (mmHg)	122.58 ± 10.04	125.18 ± 9.22 ^b^	148.29 ± 22.69 ^b,c^	151.80 ± 22.78 ^b,c,d^	<0.001
Diastolic BP (mmHg)	73.72 ± 7.36	75.89 ± 6.96 ^b^	84.23 ± 11.47 ^b,c^	87.06 ± 11.54 ^b,c,d^	<0.001
FBG (mmol/L)	5.27 ± 0.42	5.34 ± 0.42	6.06 ± 1.77 ^b,c^	6.28 ± 1.96 ^b,c,d^	<0.001
TC (mmol/L)	4.87 ± 0.91	4.98 ± 0.93 ^b^	5.30 ± 1.10 ^b,c^	5.45 ± 1.11 ^b,c,d^	<0.001
TG (mmol/L)	0.95 ± 0.32	1.06 ± 0.32 ^b^	1.66 ± 1.57 ^b,c^	2.17 ± 1.77 ^b,c,d^	<0.001
LDL-C (mmol/L)	2.62 ± 0.68	2.82 ± 0.71 ^b^	2.92 ± 0.82 ^b,c^	3.15 ± 0.86 ^b,c,d^	<0.001
HDL-C (mmol/L)	1.50 ± 0.35	1.40 ± 0.31 ^b^	1.46 ± 0.42 ^b,c^	1.30 ± 0.34 ^b,c,d^	<0.001
Creatinine (μmol/L)	69.98 ± 11.85	68.90 ± 12.47	71.77 ± 14.00 ^b,c^	72.55 ± 15.09 ^b,c,d^	<0.001
eGFR (mL/min/1.73 m^2^)	103.09 ± 29.11	103.41 ± 10.02	98.24 ± 43.18 ^b,c^	98.59 ± 23.51 ^b,c^	<0.001
Mildly reduced eGFR (%)	225 (9.0)	71 (7.0)	805 (22.6)	840 (20.7)	<0.001

Data are mean ± SD or number (%) MHNO metabolically health non-obese, MHO metabolically healthy obese, MANO metabolically abnormal obese, MAO metabolically abnormal obese, BMI body mass index, WC waist circumference, BP blood pressure, FBG fasting blood glucose, TC total cholesterol, TG triglyceride, LDL-C low-density lipoprotein cholesterol, HDL-C high-density lipoprotein cholesterol. eGFR estimated glomerular filtration rate. ^a^ Difference between four groups; ^b^ *p* < 0.05 compared with MHNO group; ^c^ *p* < 0.05 compared with MHO group; ^d^ *p* < 0.05 compared with MANO group.

**Table 3:** Logistic regression analysis of the association between different obese phenotypes and mild reduced eGFR by gender.

Characteristics	Model 1			Model 2			Model 3		
	OR (95%CI)			OR (95%CI)			OR (95%CI)		
	General	Female	Male	General	Female	Male	General	Female	Male
Female	**1.528** **(1.303, 1.975)**	-	-	**1.544** **(1.319, 1.808)**	-	-	**1.475** **(1.258, 1.729)**	-	-
MHNO	1 (reference)	1 (reference)	1 (reference)	1 (reference)	1 (reference)	1 (reference)	1 (reference)	1 (reference)	1 (reference)
MHO	1.097 (0.763, 1.575)	0.957 (0.591, 1.549)	1.297 (0.747, 2.251)	1.039 (0.695, 1.554)	1.033 (0.604, 1.768)	1.020 (0.553, 1.883)	1.107 (0.738, 1.660)	1.068 (0.622, 1.835)	1.106 (0.598, 2.047)
MANO	1.042 (0.844, 1.287)	0.925 (0.693, 1.235)	1.162 (0.852, 1.585)	1.026 (0.829, 1.269)	0.924 (0.690, 1.237)	1.130 (0.826, 1.547)	0.800 (0.601, 1.066)	0.865 (0.589, 1.269)	0.652 (0.418, 1.018)
MAO	**1.604** **(1.303, 1.975)**	1.204 (0.909, 1.596)	**2.217** **(1.624, 3.026)**	*1.496* *(1.127, 1.985)*	1.283 (0.873, 1.886)	*1.725* *(1.133, 2.627)*	1.119 (0.738, 1.660)	1.150 (0.725, 1.824)	0.935 (0.546, 1.600)
Age (years)	**1.280** **(1.266, 1.294)**	**1.304** **(1.283, 1.326)**	**1.258** **(1.239, 1.278)**	**1.279** **(1.265, 1.294)**	**1.303** **(1.281, 1.325)**	**1.260** **(1.241, 1.280)**	**1.283** **(1.268, 1.298)**	**1.308** **(1.286, 1.331)**	**1.264** **(1.244, 1.284)**
Current smoking	0.970 (0.832, 1.130)	0.949 (0.754, 1.196)	0.951 (0.775, 1.168)	0.972 (0.833, 1.133)	0.933 (0.739, 1.178)	0.968 (0.788, 1.190)	0.962 (0.824, 1.123)	0.912 (0.720, 1.155)	0.965 (0.784, 1.187)
Current drinking	**0.528** **(0.432, 0.645)**	0.691 (0.399, 1.196)	**0.511** **(0.414, 0.631)**	**0.528** **(0.432, 0.645)**	0.691 (0.399, 1.196)	**0.509** **(0.412, 0.629)**	**0.535** **(0.438, 0.653)**	0.705 (0.407, 1.223)	0.869 (0.508, 1.486)
BMI (kg/m^2^)	-	-	-	0.996 (0.959, 1.035)	0.969 (0.920, 1.02)	1.050 (0.992, 1.111)	0.994 (0.957, 1.033)	0.967 (0.918, 1.018)	1.050 (0.598, 2.047)
WC (cm)	-	-	-	1.006 (0.995, 1.017)	1.010 (0.995, 1.025)	0.998 (0.981, 1.015)	1.002 (0.991, 1.014)	1.008 (0.993, 1.023)	0.992 (0.975, 1.009)
Hypertension	-	-	-	-	-	-	1.028 (0.840, 1.258)	0.770 (0.593, 1.001)	*1.550* *(1.114, 2.158)*
Hyperglycemia	-	-	-	-	-	-	*1.247* *(1.068, 1.455)*	*1.265* *(1.025, 1.561)*	*1.260* *(1.001, 1.587)*
Dyslipidemia		-	-	-	-	-	**1.544** **(1.315, 1.814)**	**1.509** **(1.216, 1.873)**	**1.576** **(1.232, 2.016)**

Model 1: adjusted for age, gender, race (includes Han and minority like Manchu, Hui people), current smoking and drinking; Model 2: adjusted for age, gender, race, current smoking and drinking, BMI and WC; Model 3: adjusted for age, gender, race, current smoking and drinking, BMI, WC, hypertension, hyperglycemia and dyslipidemia. Italic means *p* < 0.05; Bold means *p* < 0.01.
